# Suggestions for a Family Ice-House

**Published:** 1882-12

**Authors:** 


### SUGGESTIONS FOR A FAMILY ICE-HOUSE.

A practical experience of many years enables me to modify the suggestions for "A Family Ice-House," in the November *Century.* In my opinion, the earth should not be excavated at all where the ice-house is to stand, as the lower tiers of ice would rapidly melt, and the drainage would not be so good. The floor should be simply the ground, level with the surrounding surface. In large ice-houses even, the melting is so gradual that the water soaks into the ground, drains being rarely used, since they are apt to be the means of conveying air to the ice. Where the land is low and wet, a drain is dug outside the building, say three feet from it on all sides, and is filled with loose stones. This will dispose of the surface water and drain the ice-house. In constructing the ice-house the uprights, of two by four or two by six inches, must be set about three feet apart, securely spiked to the bottom plank, and braced between. After boarding up inside and out with common boards, and filling the spaces with dry sawdust, stamping it down as it is being filled, two-inch narrow strips should be nailed perpendicularly on the outside, about six feet apart, and on these should be nailed the clap-boards. Thus an outside air space will be obtained, through which the hot air will circulate in summer, reducing the temperature. There should be a square ventilator on the roof, with open slats on each side. In packing the ice, place the cakes close to each other, and close to the sides of the building, chinking the spaces between with broken ice as each layer is completed. No sawdust should be put on before the top layer is in, and then not so much as to cause heating. Ice packed in this way will keep better, and there is less waste.—*From the Century.*

---

Public Health.—As the result of sanitary science the death rate in England has been decreased 12 per cent. in urban and 8½ in rural districts. The number who survive youth by reaching the age of twenty has been increased 12.6-10 per cent. during the last years, while the number of those who pass from twenty to thirty-five years has been increased 12.2-10 in the same period. Two hundred years ago in England, small-pox, when it became prevalent, killed 96 out of every 1,000, and 66½ in Germany. Now the mortality from this cause is less than 1 in 1,000. Sanitary science has thus saved 59 deaths in every 1,000 in England, and 66½ in Germany, and such a visitation as the black death in Europe would never be known again until sanitary science became a thing unknown. The number of deaths from typhus had been reduced in England more than half, and the same was true of any other disease. Indeed it had come to be the belief of scientific and thoughtful men that preventable sickness unprevented is a crime against society, and that preventable death unprevented is a crime against God.

Cure for Corns.—Take one-fourth cup of strong vinegar, crumb finely into it some bread. Let stand half an hour, or until it softens into a good poultice. Then apply on retiring at night. In the morning the soreness will be gone, and the corn can be picked out. If the corn is a very obstinate one, it may require two or more applications to effect a cure.

